# Clinical-radiomics nomogram for identifying HER2 status in patients with breast cancer: A multicenter study

**DOI:** 10.3389/fonc.2022.922185

**Published:** 2022-09-07

**Authors:** Caiyun Fang, Juntao Zhang, Jizhen Li, Hui Shang, Kejian Li, Tianyu Jiao, Di Yin, Fuyan Li, Yi Cui, Qingshi Zeng

**Affiliations:** ^1^ Department of Radiology, Shandong Provincial Qianfoshan Hospital, The First Hospital Affiliated Hospital of Shandong First Medical University, Jinan, China; ^2^ Postgraduate Department, Shandong First Medical University and Shandong Academy of Medical Sciences, Jinan, China; ^3^ GE Healthcare Precision Health Institution, Shanghai, China; ^4^ Department of Radiology, Shandong Mental Health Center, Jinan, China; ^5^ Department of Radiology, Shandong Provincial Hospital Affiliated to Shandong First Medical University, Jinan, China; ^6^ Department of Radiology, Qilu Hospital of Shandong University, Jinan, China

**Keywords:** breast cancer, human epidermal growth factor receptor 2, radiomics, nomogram, magnetic resonance imaging

## Abstract

**Purpose:**

To develop and validate a clinical-radiomics nomogram based on radiomics features and clinical risk factors for identification of human epidermal growth factor receptor 2 (HER2) status in patients with breast cancer (BC).

**Methods:**

Two hundred and thirty-five female patients with BC were enrolled from July 2018 to February 2022 and divided into a training group (from center I, 115 patients), internal validation group (from center I, 49 patients), and external validation group (from centers II and III, 71 patients). The preoperative MRI of all patients was obtained, and radiomics features were extracted by a free open-source software called 3D Slicer. The Least Absolute Shrinkage and Selection Operator regression model was used to identify the most useful features. The radiomics score (Rad-score) was calculated by using the radiomics signature-based formula. A clinical-radiomics nomogram combining clinical factors and Rad-score was developed through multivariate logistic regression analysis. The performance of the nomogram was evaluated using receiver operating characteristic (ROC) curve and decision curve analysis (DCA).

**Results:**

A total of 2,553 radiomics features were extracted, and 21 radiomics features were selected as the most useful radiomics features. Multivariate logistic regression analysis indicated that Rad-score, progesterone receptor (PR), and Ki-67 were independent parameters to distinguish HER2 status. The clinical-radiomics nomogram, which comprised Rad-score, PR, and Ki-67, showed a favorable classification capability, with AUC of 0.87 [95% confidence internal (CI), 0.80 to 0.93] in the training group, 0.81 (95% CI, 0.69 to 0.94) in the internal validation group, and 0.84 (95% CI, 0.75 to 0.93) in the external validation group. DCA illustrated that the nomogram was useful in clinical practice.

**Conclusions:**

The nomogram combined with Rad-score, PR, and Ki-67 can identify the HER2 status of BC.

## Introduction

Breast cancer (BC) is the most common malignancy worldwide and the main cause of cancer-related death in women (1, 2). The prognosis of BC has improved since the appearance of targeted therapies, especially for patients with a human epidermal growth factor receptor 2 (HER2)-positive subtype (3). HER2-positive BC is characterized by high invasiveness, high degree of malignancy, recurrence, and metastasis, and poor prognosis (4, 5). Therefore, accurate assessment of the HER2 status is very important for the prognosis prediction and treatment decision-making for BC patients.

At present, the HER2 status is mainly detected by immunohistochemistry (IHC) or fluorescence *in situ* hybridization (FLSH), both of which are invasive methods involving tissue samples (6). However, the consistency of the HER2 status between core needle biopsy and subsequent resection biopsy of the same BC is 81%–96% (7, 8). Therefore, the development of a non-invasive and reliable method is essential for the assessment of the HER2 status in BC patients. Magnetic resonance imaging (MRI), an essential tool in breast imaging, is considered to be one of the most sensitive imaging methods for detecting BC and monitoring neoadjuvant chemotherapy (9, 10). T2WI can be used to detect bleeding, edema, and cyst in breast lesions (11). Diffusion-weighted imaging (DWI), a common method to evaluate the micro-architecture of the tumors based on the measurement of the Brownian motion of water molecules, improves the accuracy of breast tumor diagnosis (12). Dynamic contrast-enhanced MRI (DCE-MRI), another common method to evaluate BC, can provide information on blood perfusion and microvessel distribution (13). The so-called imaging features, such as blurred boundary, irregular shape, and lobulated or burr mass, are useful for the diagnosis of BC, whereas the features have limited performance in predicting the HER2 status (14). Radiomics is a new machine learning method that aims to extract a large number of quantitative features from medical images using data characterization algorithms (15). These quantitative features have been applied to identify benign and malignant breast lesions and predict neoadjuvant chemotherapy response and lymph node metastasis (16–18).

In the present study, to identify the HER2 status of BC patients, we hypothesized that the combination of radiomics signatures and clinical factors could evaluate the HER2 status in BC patients. To verify the feasibility of our hypothesis, radiomics features were selected using the Least Absolute Shrinkage and Selection Operator (LASSO) logistic model based on the radiomics features extracted from fat suppression T2WI (FS-T2WI), DWI, and DCE-MRI. A clinical-radiomics nomogram model integrating radiomics signatures and clinical risk factors was constructed by multivariate logistic regression analysis and verified by the multicenter dataset.

## Materials and methods

### Patients

**Figure 1:**
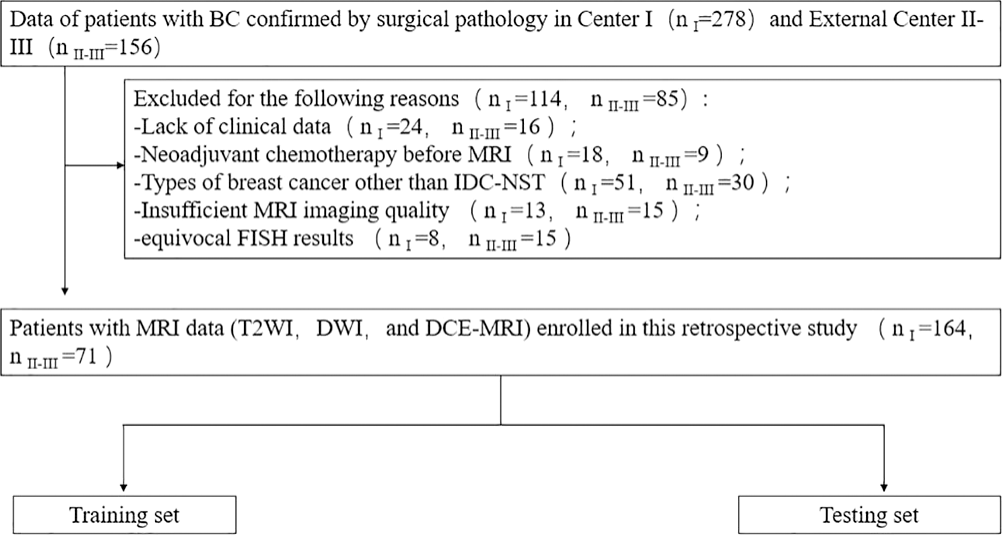
Patient recruitment routes in center I and external centers II-III. n _I_, number of patients in center I; n _II-III_, total number of patients in external centers II-III.

The retrospective study was approved by the local institutional review board, and the requirement for informed consent was waived. From July 2018 to February 2022, the MR images and pathological data of BC patients were collected from three clinical centers (center I, the First Affiliated Hospital of Shandong First Medical University; center II, Provincial Hospital Affiliated to Shandong First Medical University; and center III, Qilu Hospital of Shandong University). The inclusion criteria were as follows: (1) postoperative pathology confirmed that BC was an invasive ductal carcinoma of no special type; (2) breast MRI was performed within 2 weeks before surgery; (3) no preoperative radiotherapy or neoadjuvant chemotherapy. The exclusion criteria were as follows: (1) incomplete clinical data or insufficient MRI quality; (2) the HER2 status was not tested by IHC or FLSH after surgery, or the IHC intensity score of patient specimens was 2 +, and FLSH was not further tested. The flowchart is shown in **Figure 1**.

In addition, the following clinical information was obtained through the patient’s electronic medical record system: age, tumor diameter, tumor grade, estrogen receptor (ER), progesterone receptor (PR), Ki-67 proliferation index, HER2, and pathological axillary lymph node (ALN) metastasis status.

### Postoperative pathological assessment

The status of HER2 was detected by IHC or FLSH after operation. According to the guidelines of the American Society of Clinical Oncology/College of American Pathologists (ASCO/CAP) (6), if the IHC result was 0 or 1+, HER2 was defined as negative; if the result was 3+, it is positive; for tumors with an IHC result of 2+, further FLSH detection was required. If gene amplification occurred, it was defined as positive. For the ER/PR test, the nuclear staining of ≥1% of tumors was defined as ER/PR positive. The critical threshold of Ki-67 to 14% was set, and tumors ≥14% were defined as high expression.

### MRI acquisition and image segmentation

Breast MRI examinations were performed using a 3.0-T MRI scanner, equipped with a special breast phased-array surface coil. Patients were placed in the prone position, and the bilateral mammary glands naturally hung in the coil to fully extend the mammary glands. FS-T2WI, DWI, and DCE-MRI were sequentially obtained, and the detailed parameters of MRI acquisition are summarized in **Supplementary Table 1**.

**Figure 2:**
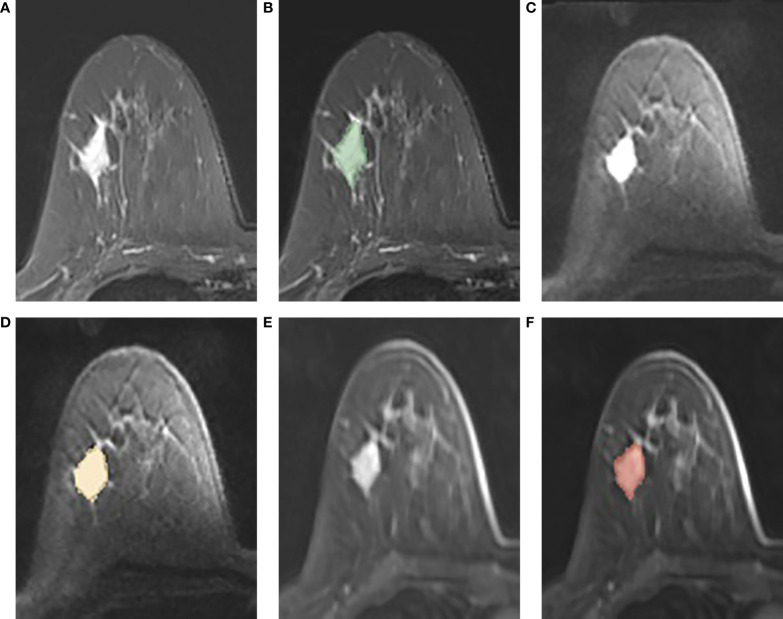
**(A-F)**: An example of manual segmentation in breast cancer. **(A, B)**: tumor area (green in fat suppression T2WI image); **(C, D)**: tumor area (orange in DWI image, b = 1,000 s/mm^2^); **(E, F)**: tumor area (red in DCE-MR image).

A free open-source software called 3D Slicer (www.slicer.org) was used to perform image segmentation. On FS-T2WI, DWI, and DCE-MRI (the peak enhancement phase of multiphase-enhanced MRI selected according to the time intensity curve), the region of interest (ROI) of each tumor was manually outlined layer by layer along the tumor contour by excluding the areas of necrosis and calcification. **Figure 2** shows an example of manual ROI drawing.

### Radiomics feature extraction and selection

Due to the difference between MRI scanning parameters and devices, we preprocessed the images before the extraction of radiomics features. We resampled the voxels of all images to 1 mm × 1 mm × 1 mm using three-line interpolation and standardized its intensity range to 0 to 255. The 3D Slicer software was also used for feature exaction according to guidelines defined by the image biomarker standardization initiative (19). Four groups of features were extracted from the FS-T2WI, DWI, DCE-MRI, and their combination (FS-T2WI+DWI+DCE-MRI).

To evaluate the intra- and interobserver agreement of feature exaction, the MR images of 30 patients were randomly selected. Two experienced radiologists (reader 1 and reader 2) blinded to clinical information completed the process manually and independently with the same criteria. Reader 1 repeated the process after 3 weeks to assess intraobserver reproducibility. The reliability of measurements was assessed by intra- and interclass correlation coefficients (ICCs). ICC values above 0.75 were considered to have good consistency, and the remaining MRI feature exaction was completed by reader 1.

Two feature selection methods, minimum-redundancy maximum-relevance (mRMR) and LASSO, were used to obtain the most significant characteristics for evaluation of the HER2 status. At first, mRMR was carried out to narrow the range of redundant and irrelevant features; 30 features were retained. Then, the retained features were filtered with LASSO to obtain the best features, and 10-fold cross validations were utilized to determine the optimal values of λ. The radiomics score (Rad-score) of each patient was calculated by selecting the linear combination of features and the product of their respective coefficients

### Model construction and validation

The seven clinical parameters (age, tumor diameter, tumor grade, ER, PR, Ki-67, and ALN metastasis status) were first analyzed by univariate logistic regression to screen out the clinical features of *P* < 0.05. To obtain the clinical risk factors identifying the HER2 status and building the clinical model, the significant variables in univariate analysis were input for stepwise multivariate logistic regression analysis. Moreover, we used multivariate logistic regression analysis to develop a clinical-radiomics model based on Rad-score and clinical risk factors, which is displayed by a nomogram.

Therefore, a total of three models were constructed to identify the HER2 status of BC: clinical model, radiomics model, and clinical-radiomics model. The area under the receiver operating characteristic (ROC) curve (AUC) was used to evaluate the discrimination performance of the three models in the training and validation cohorts. Finally, to explore the clinical utility of nomogram, decision curve analysis (DCA) was carried out based on three models to determine the utility of nomogram in a series of threshold probabilities.

### Statistical analysis

R programming language (version 4.1.0, www.programmingr.com) was applied for statistical analysis and data processing. The differences of continuous variables (age, tumor diameter) between the HER2-negative group and HER2-positive group were compared by the independent sample *t*-test or Mann–Whitney U-test and described as mean ± standard deviation (SD). The differences of categorical variables (ER, PR, Ki-67, ALN metastasis status, and tumor grade) between the two groups were compared using chi-square test or Fisher’s exact test and expressed as absolute numbers (n) and proportions (%). Univariate and multivariate logistic regression analyses were used to evaluate the relationship between HER2 overexpression status and clinical risk factors. All statistical tests were two-sided, and *P*-values of <0.05 were regarded as significant.

## Results

### Clinical characteristics

A total of 235 breast cancer patients was consecutively enrolled (met the inclusion criteria, but not the exclusion criteria). All the patients were women with the mean age of 50.76 ± 10.82 years (range: 26-83 years). The patients were divided into three independent groups: a training group (from center I, 115 patients), internal validation group (from center I, 49 patients), and external validation group (from centers II and III, 71 patients).

The clinical characteristics of the three groups were compared as shown in **Table 1**. The HER2-positive proportions in the training, internal validation, and external validation sets were 27.1%, 26.5%, and 32.4%, respectively.

**Table 1:** Patient characteristics in the training and validation cohorts (mean ± standard deviation).

Clinicopathological features	Training group (N = 115)		*P*	Internal validation group (N = 49)		*P*	External validation group (N = 71)		*P*
	HER2- (n = 84)	HER2+ (n = 31)		HER2- (n = 36)	HER2+ (n = 13)		HER2- (n = 48)	HER2+ (n = 23)	
Age (years, mean± SD)	49.5 ± 10.5	52.5 ± 8.9	0.163	52.6 ± 11.4	50.3 ± 7.5	0.494	49.8 ± 12.4	52.4 ± 11.3	0.396
Diameter (cm, mean± SD)	2.1 ± 0.9	2.2 ± 0.7	0.658	1.8 ± 0.7	2.8 ± 1.1	0.000	1.9 ± 0.8	2.4 ± 0.8	0.034
ER			0.003			1.000			0.034
Positive	72 (85.7%)	18 (58.1%)		34 (94.4%)	12 (92.3%)		37 (77.1%)	12 (52.2%)	
Negative	12 (14.3%)	13 (41.9%)		2 (5.6%)	1 (7.7%)		11 (22.9%)	11 (47.8%)	
PR			0.001			0.352			0.000
Positive	68 (81.0%)	15 (48.4%)		31 (86.1%)	9 (69.2%)		38 (79.2%)	8 (34.8%)	
Negative	16 (19.0%)	16 (51.6%)		5 (13.9%)	4 (30.8%)		10 (20.8%)	15 (65.2%)	
Ki-67			0.001			0.040			0.038
≥14%	48 (57.1%)	28 (90.3%)		20 (55.6%)	12 (92.3%)		40 (83.3%)	23 (100%)	
<14%	36 (42.9%)	3 (9.7%)		16 (44.4%)	1 (7.7%)		8 (16.7%)	0	
Pathological ALN metastasis			0.751			0.176			0.399
Positive	28 (33.3%)	12 (38.7%)		10 (27.8%)	7 (53.8%)		22 (45.8%)	13 (56.5%)	
Negative	56 (66.7%)	19 (61.3%)		26 (72.2%)	6 (46.2%)		26 (54.2%)	10 (43.5%)	
Histological grade			0.210			0.289			0.017
I	17 (20.2%)	2 (6.5%)		9 (25.0%)	0		2 (4.2%)	0	
II	51 (60.7%)	22 (71.0%)		22 (61.1%)	8 (61.5%)		38 (79.2%)	12 (52.2%)	
III	16 (19.0%)	7 (22.6%)		5 (13.9%)	5 (38.5%)		8 (16.7%)	11 (47.8%)	
Rad-score(median)	-1.3[-0.9, -0.1]	-0.7[-0.9, -0.1]	<1e-04	-1.1[-1.7, -0.8]	-0.6[-0.9, -0.3]	0.002	-1.1[-1.7, -0.8]	-0.9[-1.2, -0.8]	0.010

ER, estrogen receptor; PR, progesterone receptor; HER2, human epidermal growth factor receptor-2; ALN, axillary lymph node.

### Intraobserver and interobserver agreement for radiomics features extraction

The intraobserver ICC was 0.761 to 0.990, and the interobserver ICC ranged from 0.759 to 0.989 for evaluation of the radiomics features extraction. The results showed good consistency of feature extraction within and between observers.

### Feature selection and development of the radiomics model

A total of 851 quantitative radiomics features were extracted from each sequence, which could be summarized into the following four groups: 14 volume and shape features (2D and 3D), 18 first-order features, 75 texture features, and 744 ([18 + 75] * 8) wavelet transform features.

**Figure 3:**
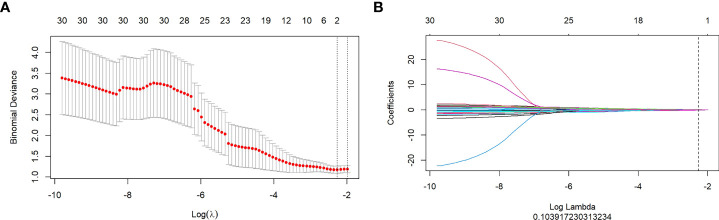
**(A, B)**: Texture feature selection using the Least Absolute Shrinkage and Selection Operator (LASSO) regression. **(A)**: Optimal tuning parameters (λ) in the LASSO model binomial deviation diagram. **(B)**: LASSO coefficient profile of the features.

By mRMR and LASSO, 6, 10, and 3 optimal radiomics features were selected from FS-T2WI, DWI, and DCE-MRI, respectively. Then, combining these three sequences, two radiomics features (one from DWI and one from DCE-MRI) were selected and executed from 2,553 (851×3) features to construct a radiomics model (**Figure 3**). The Rad-score of each patient was calculated using the formula presented in **Supplementary Materials 2**.

### Development of the clinical and clinical-radiomics models

Based on the univariate and stepwise multivariate logistic regression analyses, two clinical risk factors (PR and Ki-67) were obtained for identification of the HER2 status and were used for construction of the clinical model. In addition, logistic regression analysis showed that the Rad-score was an independent variable to identify HER2 status (**Table 2**). Therefore, a clinical-radiomics model was constructed by combining Rad-score and clinical risk factors.

**Table 2:** Univariate and multivariate analyses of risk factors for HER2.

Variable	Univariate logistic analysis		Multivariate logistic analysis	
	OR (95% CI)	*P*	OR (95% CI)	*P*
ER	0.23 [0.09, 0.59]	0.002	NA	NA
PR	0.22 [0.09, 0.53]	0.000	0.37 [0.13, 1.07]	0.067
Ki-67	7.00 [1.97, 24.84]	0.002	4.12 [0.98, 17.37]	0.053
Rad-score	11.85 [4.25, 33.02]	<1e-04	9.88 [3.43, 28.43]	<1e-04

OR, odds ratio; CI, confidence interval; NA, not available; ER, estrogen receptor; PR, progesterone receptor; HER2, human epidermal growth factor receptor-2.

### Comparison of models and establishment of clinical-radiomics nomogram

**Figure 4:**
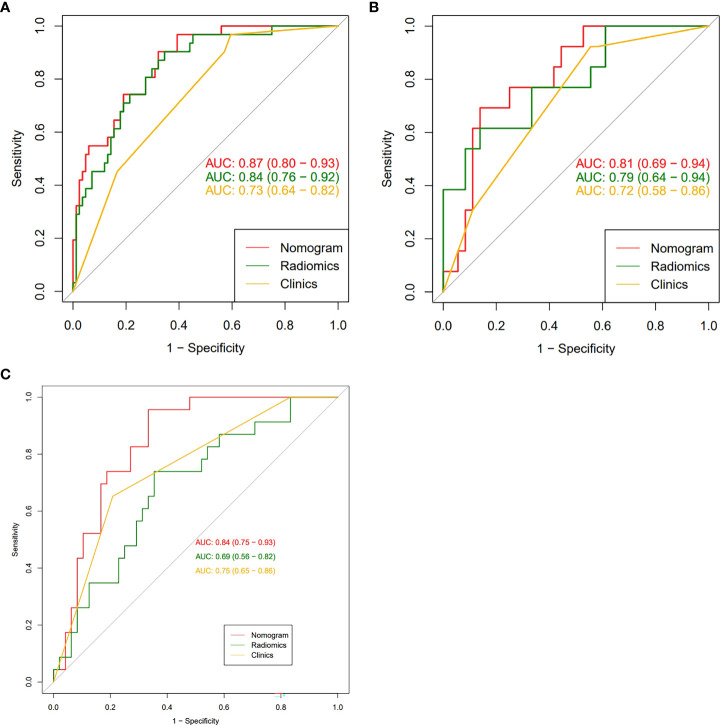
(**A-C**): The receiver operating characteristic curves of nomogram, radiomic signatures, and clinical risk factors for identifying the HER2 status of breast cancer were presented in the training group **(A)**, the internal validation group **(B)** and the external validation group **(C)**, respectively. The nomogram obtained the highest area under the curve (AUC).

**Figure 5:**
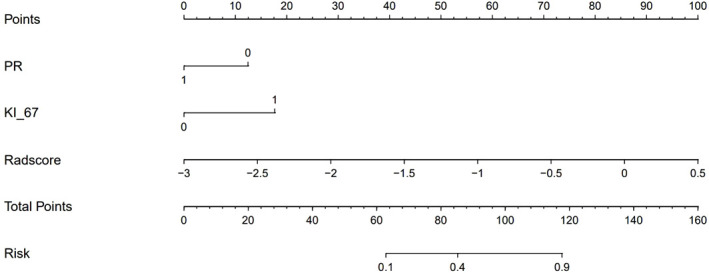
A clinical-radiomics nomogram. The nomogram was composed of Rad-score, PR, and Ki-67. PR: 0 = negative, 1 = positive; Ki-67: 0 = low expression, 1 = high expression.

To compare the performance of the clinical-radiomics model, the radiomics model, and the clinical model in identifying the HER2 status, we plotted the ROC curves of the three models (**Figure 4**). In the training cohort, the clinical-radiomics model showed the highest discrimination between HER2-negative and positive cases, with an AUC of 0.87 (95% CI, 0.80 to 0.93). The AUC value of the clinical-radiomics model was significantly higher than that of the radiomics model (AUC = 0.84, 95% CI, 0.76 to 0.92) and clinical model (AUC = 0.73, 95% CI, 0.64 to 0.82). In the internal validation and external validation cohorts, the AUC of the clinical-radiomics model was 0.81 (95% CI, 0.69 to 0.94) and 0.84 (95% CI, 0.75 to 0.93), respectively, which was superior to the single radiomics model and clinical model. The clinical-radiomics model showed the best ability to identify the HER2 status. Therefore, a clinical-radiomics nomogram was developed based on the clinical-radiomics model (**Figure 5**).

### Clinical application

**Figure 6:**
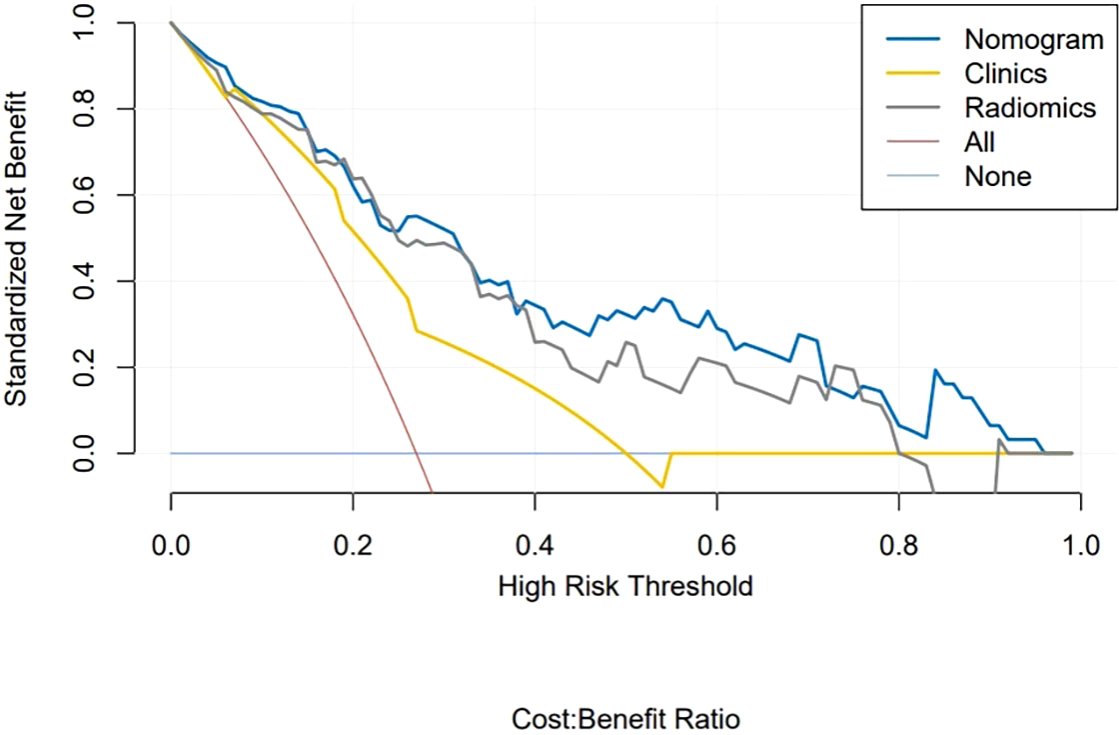
Decision curve analysis of clinical application evaluation of the nomogram. The vertical axis displays standardized net benefit. The two horizontal axes show the corresponding relationship between risk threshold and cost-benefit ratio. Compared with the radiomics signature (gray line) and clinical characteristics (yellow line), the nomogram (blue line) achieved the highest net benefit.

**Figure 6** shows the DCA curves of the clinical-radiomics model, the radiomics model, and the clinical model. According to the DCA, the clinical-radiomics nomogram showed good clinical practicability in all threshold probabilities and obtained the greatest benefit. It indicated that the nomogram was a reliable clinical tool and could be used to identify the HER2 status of BC.

## Discussion

BC is one of the most common death causes of cancer among women in the world. However, the way of BC treatment has changed drastically since HER2 is a target of the monoclonal antibody trastuzumab as well as of other anti-HER2 compounds. In this study, to identify the HER2 status in BC patients, we developed and validated a clinical-radiomics nomogram based on radiomics features and clinical risk factors. It successfully stratified BC patients according to HER2 status and performed well in the training, internal, and external validation groups.

In the present study, PR and Ki-67 were identified as clinical risk factors for distinguishing the HER2 status by multivariate logistic regression analysis. PR promotes cell growth through nuclear pathways and non-nuclear pathways. There is a negative correlation between HER2 overexpression and PR expression, which is due to the loss of the PR protein caused by HER2 overexpression through the PI3K/Akt signaling pathway (20). A previous study has shown that the expression level of PR in BC with overexpression or high amplification level of HER2 is lower than that of low-level tumor (21). Ki-67 is a nuclear protein, which is usually used to detect and quantify tumor-proliferating cells. Its increased expression is related to cell growth (22). The Ki-67 index is positively correlated with HER2 status, which indicates that HER2 overexpression may upregulate the expression of Ki-67 (23). This was consistent with our results. Based on these clinical risk factors, we further obtained the clinical model to identify the HER2 status through multivariable logistic regression analysis. The AUC in the training, internal, and external validation groups were 0.73, 0.72, and 0.75 respectively, indicating that the discrimination efficiency of the model is good.

Radiomics, a research hotspot in the field of medical imaging analysis recently, is gaining importance in the evaluation of cancer by improving tumor diagnostic, prognostic, and predictive accuracy. The advantage of radiomics is the application of a large number of automatic data feature extraction algorithms to transform image data into quantitative features. In the present study, a radiomics model for identification of the HER2 status of BC patients was constructed on the basis of the extracted features (one from DWI and one from DCE-MRI). The AUC of the constructed radiomics model was 0.79 (internal validation group), which was similar to the previous studies (24, 25). Zhou et al. (24) reported a development of radiomic features based on mammography, including mediolateral oblique and cranial caudal views, to evaluate the BC HER2 status. The best combination of the two views was achieved, and the AUC of the test set was 0.787. In another study, the features extracted from T2WI in combination with DCE-MRI showed that the ability of predicting the HER2 status of BC patients was better than that of single-parameter MRI, and the AUC of the validation set was 0.81 (25).

Accurate identification of the HER2 status plays an essential role in the evaluation of treatment options for BC patients. The use of HER2 expression as a predictive biomarker of target drug response to trastuzumab is becoming a standard recommendation for the treatment of invasive breast cancer (26). To accurately identify the HER2 status of BC patients, we further established the clinical-radiomics nomogram based on radiomics features and clinical risk factors. The performance of the nomogram in identifying HER2 status was further improved, with AUC of 0.87 in the training group and 0.81 in the internal validation group. In the present study, we used the external validation set to verify the clinical-radiomics nomogram. The results showed that it had good prediction efficiency (AUC = 0.84), and its identification ability was significantly superior than that of single radiomics features and clinical features. Our multicenter data provided additional radiomics evidence for predicting the HER2 status of BC. It can be used as a non-invasive identification tool for the HER2 status. Doctors can add the scores of each prediction index to get the total score according to the individual differences of patients, so as to make a more accurate prediction, help clinical decision-making more intuitively, and make personalized treatment plans. In addition, this study uses DCA to evaluate the clinical application of the nomogram. The DCA results show that the net benefit of the nomogram is higher than that of the radiomics model and clinical model, which increases the reliability of the model.

However, this study still has some limitations. Firstly, we only included the invasive ductal carcinoma of no special type in this study, because this pathological type accounts for 80% of all BC. This choice can avoid confounding factors associated with pathological types. Secondly, in DCE-MR images, we only selected the most obvious enhancing phase according to the time intensity curve and did not analyze the pre-contrasts and other enhanced images. Finally, this study is retrospective, and the sample size is relatively small, so some bias is inevitable. In future studies, large sample size prospective randomized studies are needed to verify the results of this study.

## Conclusions

In conclusion, combined with radiomics features and clinical risk factors, a clinical-radiomics nomogram was constructed to evaluate the HER2 status of BC patients. It can be used for identifying the HER2 status in BC patients, helping clinical decision-making, and providing supplementary information for precise medical treatment.

## Data availability statement

The original contributions presented in the study are included in the article/**Supplementary Material**. Further inquiries can be directed to the corresponding author.

## Ethics statement

The studies involving human participants were reviewed and approved by The Ethical Review Committee of The First Affiliated of Shandong First Medical University, Provincial Hospital Affiliated to Shandong First Medical University and Qilu Hospital of Shandong university. Written informed consent for participation was not required for this study in accordance with the national legislation and the institutional requirements.

## Author contributions

CF wrote the original draft preparation and design. KL, TJ, DY, FL, and YC collected the data. CF, JZ, JL, and HS analyzed the data and built the prediction models. QZ revised the manuscript. All authors contributed to the article and approved the submitted version.

## Conflict of interest

Author JZ was employed by GE Healthcare, Shanghai, China.

The remaining authors declare that the research was conducted in the absence of any commercial or financial relationships that could be construed as a potential conflict of interest.

## Publisher’s note

All claims expressed in this article are solely those of the authors and do not necessarily represent those of their affiliated organizations, or those of the publisher, the editors and the reviewers. Any product that may be evaluated in this article, or claim that may be made by its manufacturer, is not guaranteed or endorsed by the publisher.
