# Supplementary material for: Clinical-radiomics nomogram for identifying HER2 status in patients with breast cancer: A multicenter study
Source: Front Oncol. 2022 Sep 7;12:922185. doi: 10.3389/fonc.2022.922185 (PMC9490879; doi:10.3389/fonc.2022.922185)
Supplement: Supplementary file 1 [file DataSheet_1.docx]

| Hospital | Scanner | Sequence | TR/TE(ms) | FOV(mm) | Matrix | Slice  Thickness(mm) | Slice  gap(mm) | Flip  Angle | b  value | Acquisition  time(min) |
| --- | --- | --- | --- | --- | --- | --- | --- | --- | --- | --- |
| The First Affiliated of Shandong First Medical University | Philips 3.0T (Ingenia) | T2WI | 4261/75 | 280 x 351 | 328 x 252 | 4 | 0.4 | 90° | - | 2 min 59 s |
|  |  | T1WI | 527/8 | 280 x 339 | 312 x 311 | 4 | 0.4 | 90° | - | 2 min 08 s |
|  |  | DWI | 6603/66 | 340 x 255 | 112 x 91 | 4 | 0.4 | 90° | 0/1000 | 2 min 06 s |
|  |  | DCE | 3.6/1.83 | 260 x 340 | 260 x 341 | 2 | -1 | 11° | - | 7 min 55 s |
|  | Siemens 3.0T (Skyra) | T2WI | 3600/54 | 340x 330 | 380 x 380 | 4 | 0.4 | 120° | - | 3 min 20s |
|  |  | T1WI | 612/8 | 360 x 360 | 312 x 320 | 4 | 0.4 | 160° | - | 1 min 51 s |
|  |  | DWI | 6700/93 | 340 x 160 | 200 x 200 | 4 | 0.4 | 180° | 0/1000 | 2 min 54 s |
|  |  | DCE | 5.65/2.46 | 360 x 360 | 384 x 384 | 2.5 | 0.5 | 15° | - | 5 min 07 s |
| Provincial Hospital Affiliated to First Medical University | Philips 3.0T (Ingenia) | T2WI | 4495/70 | 280 x 350 | 328 x 332 | 3 | 0 | 90° | - | 3 min 34 s |
|  |  | T1WI | 527/8 | 280 x 350 | 328 x 332 | 3 | 0 | 90° | - | 2 min 59 s |
|  |  | DWI | 7011/67 | 320 x 350 | 148 x 153 | 4 | 1 | 90° | 0/1000 | 1 min 35 s |
|  |  | DCE | 4.8/2.1 | 280 x 350 | 280 x 339 | 1 | 0 | 12° | - | 6 min 57 s |
| Qilu Hospital of Shandong University | GE 3.0T (Signa HDx) | T2WI | 2984/50 | 350 x 350 | 384 x 255 | 6 | 1 | 111° | - | 1 min 29 s |
|  |  | T1WI | 723/6 | 350 x 350 | 384 x 255 | 6 | 1 | 111° | - | 1 min 39 s |
|  |  | DWI | 3000/45 | 350 x 350 | 128 x 128 | 6 | 1 | 90° | 0/1000 | 2 min 06 s |
|  |  | DCE | 3.9/1.6 | 360 x 360 | 383 x 224 | 1.8 | 0.8 | 5° | - | 7 min 08 s |

**Supplementary Table 1.** Multi-parameter MRI sequences and acquisition parameters in three centers.

Notes: FOV, field of view; TR, repetition time; TE, echo time.

**Supplementary material 2.**

The radiomics score was calculated by the following formula:

$$\boldsymbol{R}\boldsymbol{adi}\boldsymbol{omics score=}\sum_{\boldsymbol{i=1}}^{\boldsymbol{N}} \left( \boldsymbol{coef}_{\boldsymbol{i}}\boldsymbol{X}_{\boldsymbol{i}} \right)$$

Notes: Where N represent the number of the selected feature, coef_i_ is the value of non-zero coefficient of the i_th_ selected feature, X_i_ is the value of the i_th_ selected feature.

Two radiomics features (one from DWI and one from DCE-MRI) were selected from 2553 texture features by using the LASSO regression model. These features were included in the Rad-score, which is calculated by using the following formula:

"Rad-score=-0.319*DCE_wavelet_HHH_gldm_SmallDependenceLowGrayLevelEmphasis+0.06*DWI_wavelet_HHH_ngtdm_Busyness+-1.004
